# The Effect of a Triple Combination of Bevacizumab, Sodium Hyaluronate and a Collagen Matrix Implant in a Trabeculectomy Animal Model

**DOI:** 10.3390/pharmaceutics13060896

**Published:** 2021-06-17

**Authors:** Vanessa Andrés-Guerrero, Irene Camacho-Bosca, Liseth Salazar-Quiñones, Nestor Ventura-Abreu, Mercedes Molero-Senosiain, Samuel Hernández-Ruiz, Guillermo Bernal-Sancho, Rocío Herrero-Vanrell, Julián García-Feijóo

**Affiliations:** 1 Department of Pharmaceutics and Food Technology, Faculty of Pharmacy, Complutense University of Madrid (UCM), IdISSC, 28040 Madrid, Spain; vandres@ucm.es (V.A.-G.); rociohv@ucm.es (R.H.-V.); 2 Innovation, Therapy and Pharmaceutical Development in Ophthalmology (InnOftal) Research Group (UCM 920415), 28040 Madrid, Spain; 3 Ophthalmology Department, San Carlos Clinical Hospital, 28040 Madrid, Spain; camachobosca@gmail.com (I.C.-B.); malisq2014@gmail.com (L.S.-Q.); ventanes@gmail.com (N.V.-A.); merce.molero@gmail.com (M.M.-S.); samuelhernandezruiz@gmail.com (S.H.-R.); guille1111992@gmail.com (G.B.-S.); 4 Department of Immunology, Ophthalmology and ORL, Instituto de Investigaciones Oftalmológicas Ramón Castroviejo, Faculty of Medicine, Complutense University of Madrid (UCM), IdISSC, 28040 Madrid, Spain

**Keywords:** intraocular pressure, trabeculectomy, anti-VEGF, bevacizumab, sodium hyaluronate, collagen implant, drug release, inflammation

## Abstract

Currently available anti-scarring treatments for glaucoma filtration surgery (GFS) have potentially blinding complications, so there is a need for alternative and safer agents. The effects of the intrableb administration of a new combination of the anti-VEGF bevacizumab, sodium hyaluronate and a collagen matrix implant were investigated in a rabbit model of GFS, with the purpose of modulating inflammation, angiogenesis, fibroblast migration and fibrogenesis in the wound healing process. A comparative-effectiveness study was performed with twenty-four rabbits, randomly assigned to the following treatments: (a) biodegradable collagen matrix implant (Olo), (b) bevacizumab-loaded collagen matrix implant (Olo-BVZ), (c) bevacizumab-loaded collagen matrix implant combined with sodium hyaluronate (Olo-BVZ-H5) and (d) sham-operated animals (control). Rabbits underwent a conventional trabeculectomy and were studied over 30 days in terms of intraocular pressure and bleb characterization (height, area and vascularity in central, peripheral and non-bleb zones). Histologic differences among groups were further evaluated at day 30 (inflammation, total cellularity and degree of fibrosis in the area of surgery). Local delivery of bevacizumab (Olo-BVZ and Olo-BVZ-H5) increased the survival of the filtering bleb by 21% and 31%, respectively, and generated a significant decrease in inflammation and cell infiltration histologically 30 days after surgery, without exhibiting any local toxic effects. Olo-BVZ-H5 showed less lymphocyte infiltration and inflammation than the rest of the treatments. Intraoperative intrableb implantation of bevacizumab, sodium hyaluronate and a collagen matrix may provide an improved trabeculectomy outcome in this model of intense wound healing. This study showed an effective procedure with few surgical complications and a novel combination of active compounds that offer new possibilities to improve the efficacy of filtration surgery.

## 1. Introduction

Glaucoma filtration surgery (GFS) often fails to adequately control intraocular pressure (IOP) caused by the persistent wound healing process, which blocks the surgically created outflow pathway at the conjunctival and episcleral plane. Diverse molecular and cellular processes, such as collagen deposition, angiogenesis and the activation and proliferation of fibroblasts, factor into in this obstruction, which subsequently increases IOP and promotes the patient’s visual deterioration. New therapeutic approaches are under investigation to improve the healing response in filtration surgery.

In GFS, bleb failure often occurs on the third to fifth postoperative day, when the maximum proliferation of subconjunctival fibroblasts occurs [1]. To minimize this biological process, biodegradable spacers such as porous collagen matrices are used in clinical practice to prevent the adhesion of ocular tissues and modify the patterns of fibroblast migration, directing wound healing away from scar formation [2]. A complementary approach involves the use of vascular endothelial growth factor inhibitors (anti-VEGFs) in GFS, since several studies have shown an improvement in surgical outcomes in humans and animals [3,4,5]. However, taking the wound healing process timeframe into account, the short half-life of anti-VEGF agents compromises their use in GFS by limiting the bioavailability of the drug in the filtering bleb. In this regard, the use of systems that are able to provide a prolonged drug effect could address this significant inconvenience.

In GFS, subconjunctival viscous solutions of sodium hyaluronate (HA) have been used to provide mechanical support for the filtering bleb and to reduce bleb failure [6]. HA can be injected through a fine-gauge needle (i.e., 30 G), remaining at the administration site and disappearing via degradation one to two weeks later [7]. An interesting application of HA in drug delivery has allowed the controlled and localized release of several ophthalmic drugs such as hypotensive agents [8,9,10], antibiotics [11] or corticoids [12], among others [13].

In the present work, we sought to evaluate the effect of an intrableb system composed of a triple combination of the anti-VEGF bevacizumab, a biodegradable collagen matrix and sodium hyaluronate, with the aim of modulating inflammation, angiogenesis, fibroblast migration and fibrogenesis in the wound healing process. This system would help maintain the space in the filtering bleb, prevent the migration and alignment of fibroblasts and prolong the presence of the anti-VEGF, allowing a longer onset of the action of bevacizumab. To our knowledge, this is the first study that shows the effect of a biodegradable intrableb system with these characteristics that is able to control various phases of the healing process, with the objective of determining whether this triple combination is able to increase the success rate of glaucoma surgery through a synergistic effect. The in vivo evaluation was carried out in a trabeculectomy rabbit model and all eyes underwent histopathological examination after the end of the clinical follow-up.

## 2. Materials and Methods

### 2.1. Preparation of the Intrableb System

The optimization of the preparation method was performed with a model protein marked with fluorescein (HSA-FITC, Sigma-Aldrich, Madrid, Spain). The protein was diluted in an isotonic buffer solution at the same concentration employed with bevacizumab (25 mg/mL). The collagen matrix employed consisted of a dry form of the scaffold that contained a connected porous structure of 10–300 µm in diameter made of crosslinked lyophilized porcine type I atelocollagen (≥90%) and glycosaminoglycan (≤10%). The collagen matrix density was 35.0 ± 7.0 mg/cm^3^, with a pH value of 7 ± 0.5 (Ologen, Aeon Astron Corporation, Taipei, Taiwan) [14]. Different volumes of HSA-FITC (25–150 µL) were added on top of the lyophilized matrix and the protein was allowed to diffuse and distribute for 30 min. HSA-FITC distribution in the matrix was analyzed by mean of optic microscopy. The protein was distributed in the porous structure, and was absorbed by the lyophilized matrix as it was being soaked.

**Figure 1:**
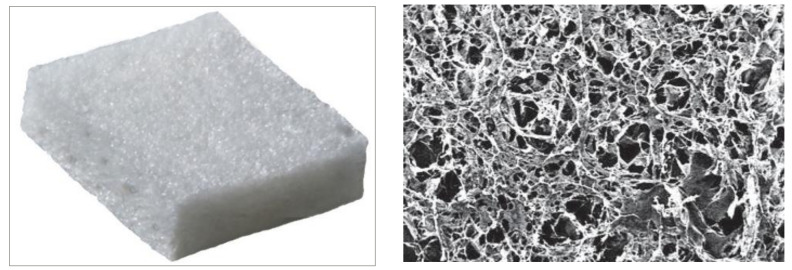
On the left, a picture of the collagen matrix implant employed (5 × 5 × 2 mm). On the right, scanning electron microscopy image showing the three-dimensional porous structure of the implant (pore diameters between 10 and 300 µm; photo credit: Aeon Astron Inc., (Leiden, The Netherlands).

**Figure 2:**
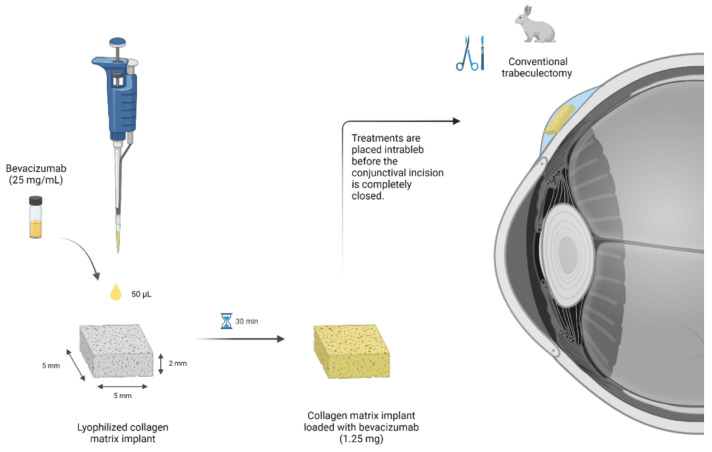
Schematic overview of the process used to load the collagen matrix implant with bevacizumab (1.25 mg), to be placed intrableb in a trabeculectomy animal model in rabbits. Created with BioRender.com (accessed on 16 June 2021).

Systems were always prepared on the day of surgery under sterile conditions in a laminar flow hood. A 10 × 10 × 2 mm sterile matrix was cut into four identical square pieces that measured 5 × 5 mm and 2 mm in height (Figure 1). Then, 1.25 mg bevacizumab (50 µL Avastin; Roche GmbH, Grenzhach-Wyhlen) were evenly distributed on each matrix, following the procedure described above. The last step was carried out during the surgical procedure once the BVZ-loaded matrix was placed into the subconjunctival space. Five microliters of sodium hyaluronate 2.3% (115 µg sodium hyaluronate 5000 KDa; 7,000,000 cps) were injected with a 25 G cannula (Healon 5; Abbott, Santa Ana, CA, USA) in the appropriate treatment group before the conjunctival incision was completely closed, completely covering the matrix. Figure 2 shows a schematic overview of the procedure.

### 2.2. Animals and Anesthesia

Twenty-four healthy New Zealand rabbits, weighing between 3 and 3.5 kg were used. Animals were kept in individual cages with food and water ad libitum under controlled cycles (12/12-h light/dark). Animal studies were approved by the Experimental Animal Ethical Committee of the San Carlos Clinical Hospital (15/017-II, 16 December 2015) and the Regional Ministry of Environment and Territory (PROEX 410/15, 13 January 2016). Furthermore, animal manipulations followed institutional guidelines, European Union regulations for the use of animals in research and the statement of the Association for Research in Vision and Ophthalmology for the use of animals in ophthalmic vision research.

### 2.3. Glaucoma Filtration Surgery (GFS)

GFS was based on a standard protocol and performed by the same surgeon (JGF) in different sessions. Prior to surgery, rabbits were anesthetized with an intramuscular injection of ketamine (20 mg/kg) and medetomidine (0.1 mg/kg). During surgery, topical ocular anesthesia was induced with 0.1% tetracaine and 0.4% oxibuprocaine eye drops (Double Anaesthetic Colircusi, Alcon, TX, USA). All animals were constantly monitored during the procedure and recovery was performed by an experienced veterinary specialist (MCRB).

Both eyes of the animals underwent a conventional trabeculectomy. To this end, a lid speculum was placed and a supertemporal, limbal-based conjunctival flap was made with Westcott scissors. The conjunctiva was bluntly dissected forward using a cotton-tip applicator. A 3 × 3 mm sclerectomy and a peripheral iridectomy were then performed. The scleral flap was repositioned and closed with two interrupted 9-0 monofilament nylon sutures (BBraun, Barcelona, Spain). The conjunctival incision was closed with a running 8-0 absorbable suture of polyglycolic acid (BBraun). Treatments were placed intrableb before the conjunctival incision was completely closed. Neomycin and prednisolone were applied topically after surgery and continued to be applied in all eyes for the first week, to control postoperative inflammation and prevent ocular infections (twice a day during the first 3 days and then once a day until day 7). Eyes were randomly assigned to the following four groups (*n* = 12 eyes/group): (1) Olo treatment group: biodegradable collagen matrix implant; (2) Olo-BVZ treatment group: biodegradable collagen matrix implant loaded with 1.25 mg bevacizumab; (3) Olo-BVZ-H5 treatment group: biodegradable collagen matrix implant loaded with 1.25 mg bevacizumab and combined with 5 µL sodium hyaluronate; and (4) sham-operated animals (control).

### 2.4. Clinical Follow-Up

The preoperative evaluation included IOP measurements (Tonovet, Icare, Vantaa, Finland) and a complete slit-lamp examination (SL-17, Kowa, CA, USA). The postoperative follow-up visits were scheduled on days 1, 2, 4, 6, 8, 9, 10, 11, 12, 13, 14, 17, 20, 23, 27 and 30, and included IOP measurements, clinical examination and bleb grading in every visit. Masked clinicians using a well-defined set of procedures ensured the most accurate evaluation in every visit.

Rabbits were kept calm for at least 5 min before IOP determinations. At any sign of stress, measurements were postponed for another 5 min. To avoid any bias arising from diurnal IOP variation, all measurements were made at a similar time of day (1.30 p.m. ± 1 h). Conjunctival hyperemia, conjunctival edema, cornea and anterior chamber were evaluated according to the following scale [15]: (a) conjunctival hyperemia or edema: normal = 0, mild = 1, moderate = 2, intense = 3; (b) cornea: normal = 0, iris details = 1, iris slightly obscured = 2, pupil barely discernible = 3, opaque = 4; (c) presence of cells in the anterior chamber: iris clearly visible = 0, iris slightly obscured = 1, pupil barely discernible = 2, iris not discernible = 3.

The external structure and surrounding conjunctival tissue of each bleb were evaluated using the Moorfields Bleb Grading System [16]. This system includes well-defined parameters that allow the estimation of area, height and vascularity of various parts of the bleb. The central demarcated area of the bleb is compared with that of the total conjunctival area. The score depends on its extension: 1 = 0% even in a small demarcated area; 2 = 25%; 3 = 50%; 4 = 75%; 5 = 100%. The whole, maximal area of the bleb is compared with that of the total area visible of conjunctiva. The score depends on its extension: 1 = 0%, 2 = 25%, 3 = 50%, 4 = 75%, 5 = 100%. Height is scored from 1 (low) to 4 (high). Vascularity in three places of the bleb is also evaluated: non-bleb conjunctiva (conjunctiva, which is more than 2 mm from the bleb edge), peripheral vascularity (in the edge) and central vascularity (in the center) of the bleb, graded from 1 (avascular) to 5 (severe vessel inflammation).

A Kaplan–Meier survival curve was drawn according to the survival time of the filtering blebs in each group (30 days of observation time). The primary outcome measure was the rate of surgical failure, defined as an IOP reduction of less than 20% from baseline. The failure of a filtering bleb was defined as a flat, neovascularized and scared filtering bleb with a deep anterior chamber.

### 2.5. Histological Analysis of Ocular Tissues

On day 30 after surgery, the rabbits were killed by means of an anesthetic overdose, and both eyes were enucleated and fixed in neutral buffered 10% formaline (Merck KGaA, Darmstadt, Germany). Eyeballs were cut in half on their long diameter with a blade using suture points as a reference, to include operated and non-operated areas (opposite limbal region). Then, both parts were embedded in paraffin wax and sequential 5-µm sections were cut following the antero-posterior axis, in order to obtain a horizontal plane of the eyes, and stained with hematoxylin and eosin. Sectioning, staining and examining of samples were carried out by a masked anatomopathologist at the Pathological Anatomy Service of the San Carlos Clinical Hospital in Madrid (ACV).

The tissue remarks noted the presence of external and internal sutures (graded from 0 to 1; 0: absence, 1: presence), fibrosis (graded from 0 to 1; 0: absence, 1: presence), inflammation (graded from 0 to 1; 0: absence, 1: presence) and, if inflammation was confirmed, it was also graded from 1 to 3 (1: slight, 2: moderate, 3: high). Plasma cells were only identified in a qualitative way (presence or absence). The presence of lymphocytes, eosinophils and giant cells was determined in a quantitative manner. Cell counts from images were made in three non-overlapping fields in the surgery site and in the contralateral site, at a magnification of 40× (*n* = 3 eyes). All the data were obtained using an Eclipse microscope (TS100F Nikon; Tokyo, Japan) at a magnification of 4×, 10×, 20× and 40× with a digital camera (Progres C3, Jenoptik, ProgRes CapturePro 2.10.0.0, Jena, Germany).

### 2.6. Statistical Analysis

All the information was analyzed with Microsoft Office Excel (2010, Microsoft, Redmond, WA, USA) and Statgraphics Centurion (v. 18, Statgraphics Tecnologies Inc., The Plains, VA, USA). For the analysis of association between variables, we employed either the χ^2^ (Chi-squared) test (qualitative variables) or Student’s *t*-test (to compare means between dichotomic quantitative variables). For the aim of the present study, the statistical significance was settled at *p*-values < 0.05. Survival curves according to the Kaplan–Meier method were plotted, and significance was tested using the log-rank test. Mean scores of histologic parameters for each treatment group were calculated, and these semiquantitative data were analyzed using analysis of variance.

## 3. Results

### 3.1. Preparation of the Intrableb System

The optimization of the preparation method, performed with a model protein marked with fluorescein (HSA-FITC), showed that the optimum volume that allowed a homogeneous distribution of the protein, without overloading the matrix, was 50 µL. Consequently, for the in vivo studies, the collagen matrix implant was loaded with 50 µL of bevacizumab (25 mg/mL).

### 3.2. In Vivo Studies in Rabbits

All the animals were comparable in terms of age, sex and initial IOP. Except for one that did not survive surgery, all of the rabbits completed four weeks of the follow-up period. The only intraoperative complication was anterior chamber hemorrhage in 16% of eyes. The most significant complication on the first postoperative day was hyphema (8% of eyes). A case of hypostagma (2% of eyes) was also reported. These conditions fully resolved 48–72 h later. No complications such as wound leakage, encysted bleb or corneal opacity were observed during the study.

#### 3.2.1. Intraocular Pressure Reduction

**Figure 3:**
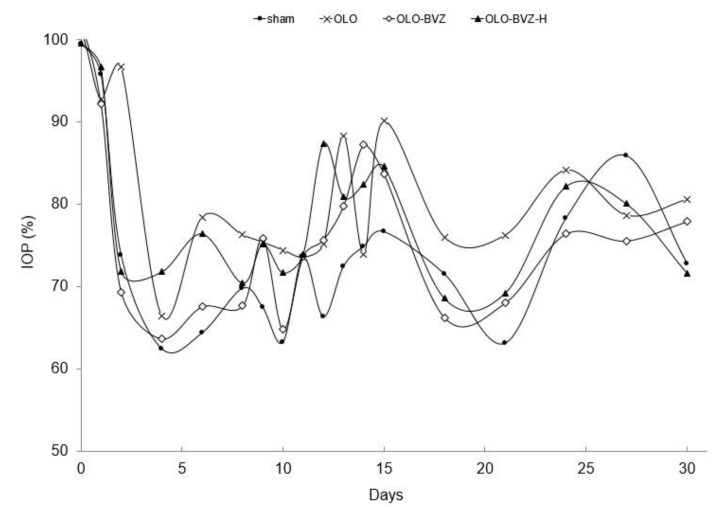
Intraocular pressure reduction (%) after surgery for all treatment groups up to 30 days. Postoperative mean IOP was significantly lower than preoperative IOP in all cases for at least 30 days. Sham-operated eyes (•), biodegradable collagen matrix implant (x), biodegradable collagen matrix implant loaded with bevacizumab (◊) and biodegradable collagen matrix implant loaded with bevacizumab, combined with sodium hyaluronate (▲).

Figure 3 shows the mean intraocular pressure (IOP) reduction as a percentage, at each follow-up after surgery for all treatment groups. The mean IOP at baseline for each group is summarized in Table 1. Postoperative IOP was significantly lower than preoperative IOP in all cases for at least 30 days. The average decrease in postoperative IOP compared to preoperative IOP (1–30 days) was 4.5 ± 0.4 mm Hg in sham-operated eyes, 5.9 ± 0.6 mm Hg in Olo, 4.6 ± 0.4 mm Hg in Olo-BVZ and 4.8 ± 0.2 mmHg in Olo-BVZ-H5 groups. At the end of the follow-up period, 33% of cases showed a mean IOP decrease of more than 20% in the control group, 67% in Olo, 67% in Olo-BVZ and 58% in Olo-BVZ-H5. No significant differences were found between IOP reductions in control and treatment groups (*p* = 0.794).

#### 3.2.2. Bleb Survival and Characteristics

**Figure 4:**
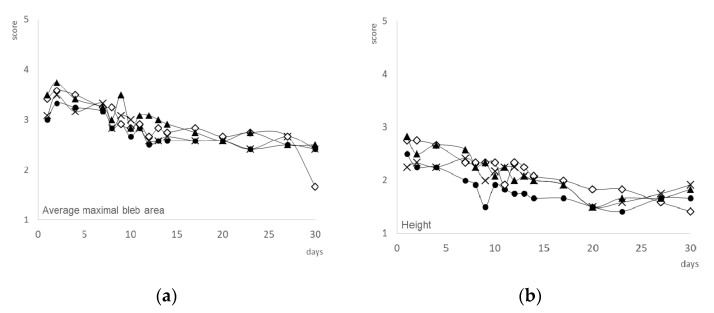

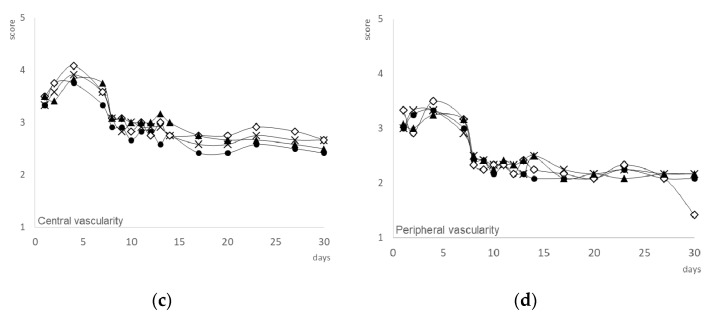
(**a**) Mean maximal area of the bleb, (**b**) height, (**c**) central vascularity and (**d**) peripheral vascularity (non-bleb) in each treatment group, compared to that of the total area visible of the conjunctiva. Groups: sham-operated animals (control •), collagen matrix (Olo group—x), bevacizumab-loaded collagen matrix (Olo-BVZ group—◊) and bevacizumab-loaded collagen matrix combined with sodium hyaluronate (Olo-BVZ-H5 group—▲). Maximal area of the bleb score: 1 = 0%, 2 = 25%, 3 = 50%, 4 = 75% and 5 = 100%. Height of the bleb in each treatment group applied to the highest point of the bleb: Score from 1 (low) to 4 (high). Vascularity of the central area of the bleb and peripheral vascularity evaluated according to the following score: 1—avascular bleb, 2—normal vascularization, 3—mild vessel inflammation, 4—moderate vessel inflammation; 5—severe vessel inflammation.

Bleb characteristics in the four groups of filtering blebs are represented in Figure 4. Table 2 shows postoperative measurements of bleb vascularity (central and peripheral) as numbers of cases and percentages at 24 h, 14 days and 30 days after surgery.

**Figure 5:**
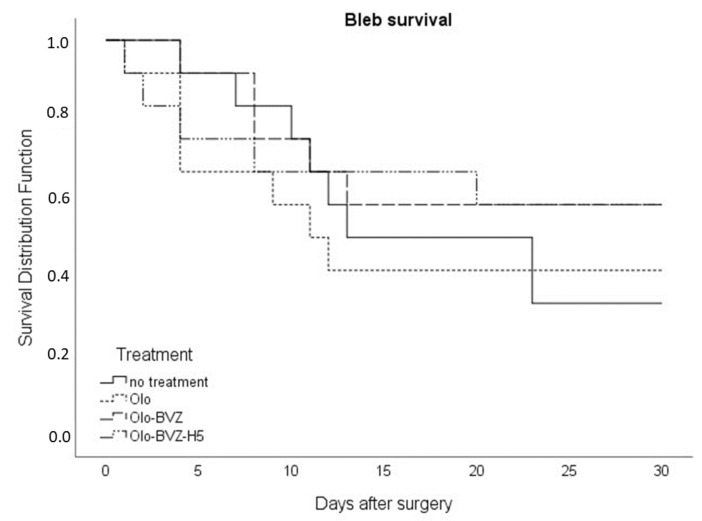
Kaplan–Meier bleb survival plot of eyes in each treatment group (*n* = 12). For each situation, bleb failure was declared when the intraocular pressure reduction was lower than 20% in two consecutive follow-up visits.

**Figure 6:**
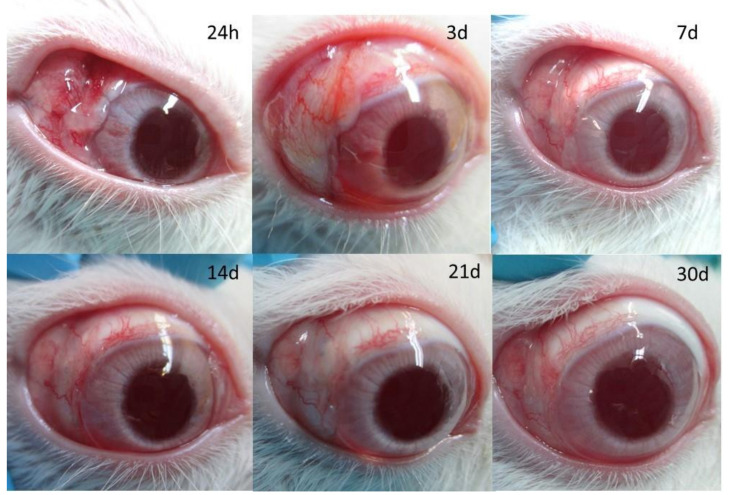
Macroscopic postoperative photographs that show surviving conjunctival blebs that remained elevated in eyes treated with bevacizumab-loaded collagen matrix combined with sodium hyaluronate (Olo-BVZ-H5). The image shows the blebs at different times after surgery (24 h, 3 d, 7 d, 14 d, 21 d and 30 d).

During the first week, moderate vessel inflammation was shown in all groups. Then, it was significantly reduced to mild during the following days in all cases (Table 2). There were no statistically significant differences in the Moorfields Bleb Grading System among the groups with regard to central bleb area, central vascularity, peripheral vascularity and bleb height at the 30-days follow-up visit. The Kaplan–Meier survival curve is shown in Figure 5. Bevacizumab showed a tendency to improve the outcome of filtration surgery in this model; however, this trend did not reach statistical significance based on the log-rank test (*p* = 0.678). The mean number of survival days were 18.6 ± 2.8, 16.2 ± 9.6, 21.2 ± 3.0 and 20.4 ± 3.5 days, for the control, Olo, Olo-BVZ and Olo-BVZ-H5, respectively. A representative eye from the group treated with bevacizumab-loaded collagen matrix implant combined with sodium hyaluronate (Olo-BVZ-H5) is shown in Figure 6.

#### 3.2.3. Histopathological Features

**Figure 7:**
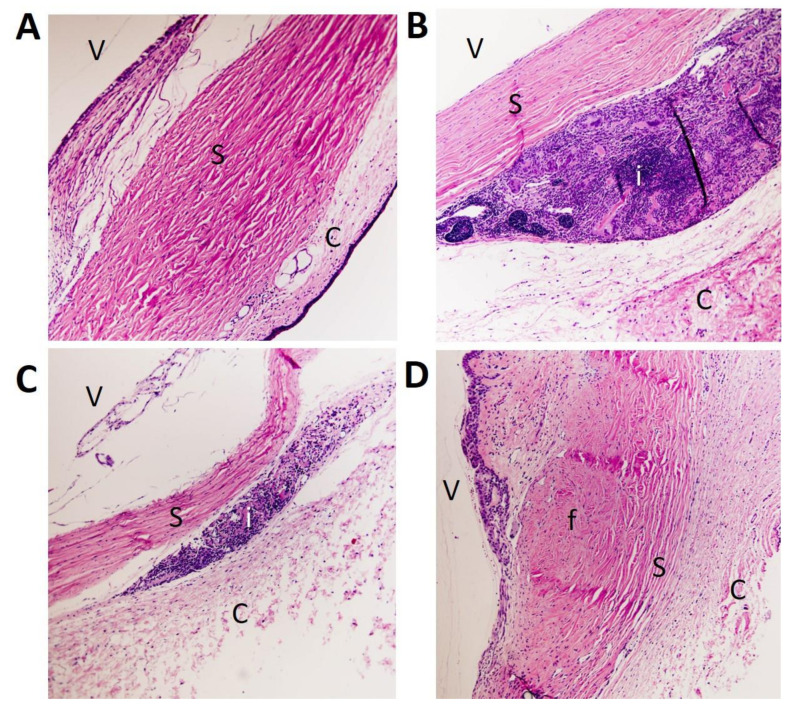
Histological view of the wound area. Hematoxylin-eosin stained sections representative of each group: (**A**) sham-operated eyes (control), **(B**) collagen matrix implant, (**C**) bevacizumab-loaded collagen matrix implant and (**D**) bevacizumab-loaded collagen matrix implant combined with sodium hyaluronate. The images were taken using a 10× magnification objective. In each picture, conjunctiva (C), sclera (S) and the vitreous (V) are indicated. (i) indicates noticeable inflammation and (f) indicates fibrosis.

**Figure 8:**
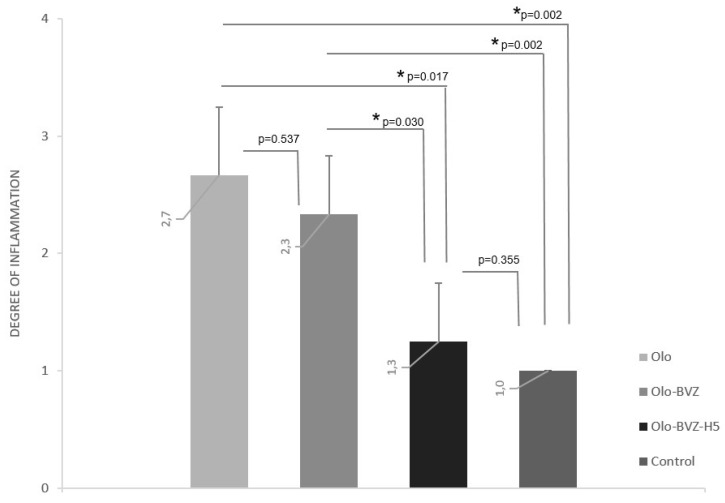
Inflammation degree, indicated by H&E staining, according to the following scale: 0—absent, 1—mild, 2—moderate and 3—severe. The analysis showed the highest inflammation in the Olo group, treated with the collagen matrix implant. * *p* < 0.05.

**Figure 9:**
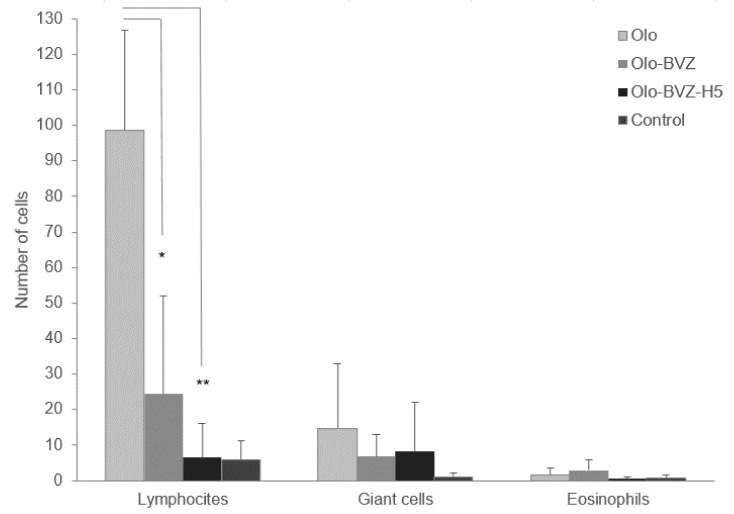
Quantitative analysis of the microscopy pictures of H&E staining showed infiltration lymphocytes at day 30 after surgery in all treatment groups (Olo, Olo-BVZ and Olo-BVZ-H5). This accumulation was higher in the group treated only with the collagen matrix implant (Olo). * *p* < 0.05, ** *p* < 0.001.

At the end of the study, histologic H&E staining was performed to evaluate microscopically whether the intrableb treatments employed affected inflammation, fibrosis and/or total cellularity in the filtering blebs. Figure 7 shows a histological view of the wound area for each group. According to the results (Figure 8), the Olo group showed the highest degree of inflammation (2.7 ± 0.6). Inflammation decreased when bevacizumab was added to the treatment, although significantly lower differences were only achieved with the combination Olo-BVZ-H5 (1.3 ± 0.6, *p* = 0.017), which demonstrated an inflammation degree that was similar to controls (*p* = 0.355). Figure 9 shows the density of lymphocytes, giant cells and eosinophils on day 30 after surgery. The addition of bevacizumab or its combination with sodium hyaluronate to the collagen matrix implant resulted in less infiltration of lymphocytes compared with the Olo group: a 4-fold and 8.5-fold reduction, respectively. No significant differences in the number of giant cells or eosinophils could be detected between treated and control eyes at the filtration site.

Considering this information together, eyes treated with the collagen matrix implant showed the highest inflammation and cellularity. Treatments that included bevacizumab considerably reduced these parameters. The triple combination of bevacizumab, sodium hyaluronate and collagen matrix (Olo-BVZ-H5) showed less inflammation and less lymphocyte infiltration among the treatment groups. Sham-operated eyes showed less inflammation and less cell infiltration (lymphocytes, giant cells and eosinophils) in all cases.

## 4. Discussion

The degree of postoperative wound healing and the amount of scar tissue formation in glaucoma filtration surgery (GFS) are critical for its success. In GFS, a fistula is created in the anterior chamber through the excision of tissue anterior to the base of the scleral flap. This trauma activates hemostasis, followed by three overlapping stages: Inflammation, proliferation and remodeling [17,18], which start immediately after the initial surgical trauma. Fibroblasts are critical in all three phases, playing a key role in the deposition of extracellular matrix (ECM) components, wound contraction and remodeling of the new ECM [19], eventually causing scarring and closure of the filtering bleb, and subsequently bleb failure. Different studies have demonstrated that fibroblast movement is directed by the orientation of the ECM, in a phenomenon known as “contact guidance” [20,21]. Although they migrate more easily on fibronectin gels, this process is hampered on collagen gels [22]. Vascular endothelial growth factor (VEGF) is a potent inducer of angiogenesis, known to have a direct effect upon the activity of fibroblasts to promote their migration, as well as the migration of inflammatory cells [23]. Based on this, the adjunctive use of VEGF inhibitors or biodegradable spacers has been tried in GFS [3].

The route of administration of anti-VEGFs in GFS is an important aspect to take into account when considering their efficacy. Although intravitreal injections have been demonstrated to be more effective in rabbits, subconjunctival anti-VEGFs have shown a longer half-life in both the iris/ciliary body and/or the retina/choroid [24]. This can be explained by the storage effect afforded by the scleral tissue matrix and the direct modulation of the conjunctival wound healing process offered by this route. In the current study, a low dose of bevacizumab was administered intrableb (1.25 mg) and combined with sodium hyaluronate and a collagen matrix implant with the aim of modulating inflammation, angiogenesis, fibroblast migration and fibrogenesis in the wound healing process. This combination allowed us to: (a) localize the delivery of the anti-VEGF in the filtering bleb, (b) provide a longer onset action of bevacizumab in the wound healing process, and (c) maintain the space in the filtering bleb while directing the pattern migration of fibroblasts.

Taking into consideration the drug release patterns of bevacizumab from the system proposed here, several processes are initiated when water penetrates the collagen matrix. The presence of enzyme dissolved in the penetrating fluid forms an enzyme–substrate complex, which later breaks up and generates a change in the matrix microstructure, affecting drug diffusion and matrix degradation itself. In the literature, several attempts to develop effective models including these phenomena can be found [25]. In the current study, sodium hyaluronate was also used to control and localize the release of bevacizumab in the bleb. Therefore, we suggest that solute diffusion and material degradation are the main driving forces for the transport of bevacizumab from the matrix, which would be released in two phases. The first phase would probably be determined by drug diffusion through the viscous hydrogel and/or the porous structure, and the second phase with the biodegradation of sodium hyaluronate and/or the collagen matrix implant.

The wound-healing response in the rabbit compared with humans is known to be more aggressive and exaggerated and to entail the routine failure of trabeculectomy surgical sites within a few days to weeks [26]. In this model of intense wound healing, we have demonstrated that the use of a low dose of bevacizumab can significantly reduce the inflammation degree and the presence of lymphocytes in the filtering bleb. It is worth mentioning the use of topical prednisone postoperatively. It is possible that this drug might interfere with the effects described for the treatments used. Nonetheless, although the corticoid quickly diffuses and distributes in the ocular tissues, the drug was applied during the first week after surgery. With an elimination half-life of about 3 h, it is therefore not very likely that the reported results at day 30 were influenced by the use of the corticoid at the beginning of the follow-up period.

As in previously published studies by other authors [27], in this work there were no remarkable differences in the mean IOP reduction among the study groups, partly because the preoperative IOPs were low in the normotensive animals used (11 mmHg), which hindered the hypotensive effect generated in this rabbit model. Thus, the primary outcome measure of bleb failure was defined as cases in which IOP reductions were 20% below baseline. At day 30, the number of cases in this situation was higher in bevacizumab-treated eyes (67% and 58% for Olo-BVZ and Olo-BVZ-H5, respectively) in comparison with controls (33% of cases), although no statistical significance could be reported.

The use of a collagen matrix implant as a bevacizumab depot in humans has been recently reported by Lommatzsch et al. in patients in which trabeculectomy was performed with mitomycin C (MMC) [28]. Authors compared the use of MMC with the use of MMC combined with a collagen matrix implant, or with a collagen matrix implant and bevacizumab 1.25 mg, infused immediately before implantation. The greatest success rate was reported in the group with only MMC (IOP < 15 mmHg without antiglaucoma medication) (72.5%), followed by the groups with the collagen matrix implant (67.5%) and the collagen matrix implant with bevacizumab (63.6%), with no significant differences found among groups. However, as the authors stated in their manuscript, these results were limited to retrospective data collection without randomization, examinations were not standardized and it was not possible to follow up the full number of eyes after 12 months of study, which could have led to a change in the significance level of these results. In any case, it has been suggested that MMC is more effective than bevacizumab in reducing IOP and achieving a diffuse filtering bleb in primary trabeculectomy. An explanation of this phenomenon involves the direct toxicity that MMC produces over the ciliary epithelium, which might decrease aqueous humor secretion and, subsequently, IOP. A toxic effect of MMC on corneal endothelial cells has been described as well, when it is administered following filtration procedures. Consequently, the use of an anti-VEGF drug, and specifically bevacizumab, may be a safer option with regard to toxicity.

Surprisingly, the wound healing response in sham-operated animals was milder than expected. These animals showed smaller and lower blebs than bevacizumab-treated eyes and a vascularization close to normal in the third week after surgery. Additionally, the histologic view of the wound area of sham-operated eyes revealed reduced cell infiltration and inflammation. It is known that the degree of scarring is determined by the severity of the initial insult and the host’s wound-healing response. Accordingly, results presented here in sham-operated eyes could be explained by the minimal tissue manipulation during surgery, which may potentially allow for a better recovery, when comparing the sham procedure with the intrableb implantation of the collagen matrix and the viscous solution of sodium hyaluronate. However, it is also important to consider that the collagen matrix implant itself might induce an inflammatory reaction even after the biomaterial is biodegraded, as previously reported by other authors [29,30]. That would also be in accordance with the inflammation reported for the treatment groups in comparison with sham-operated eyes. Given the success of surgery in the bevacizumab-treated eyes, we were surprised to find that in the clinical follow-up there was no difference in vascularity grading between groups. These results could be explained, in part, if we consider that the effect generated by this system was mediated to a greater degree through the blockade of the inflammatory signaling, regulated by VEGF, rather than by its antiangiogenic properties, as was revealed by the histologic studies.

All ocular surgeries elicit cellular and tissue responses, such as inflammation, wound healing, foreign body reactions and fibrous encapsulation, which are usually enhanced when biomaterials are locally implanted. Macrophages and their fused morphologic variants, the multinucleated giant cells, are the dominant early responders to biomaterial implantation and remain at biomaterial–tissue interfaces for the lifetime of the system. An essential function of these cells is to mediate the degradation of bioresorbable materials through extracellular degradation and phagocytosis [31]. Additionally, antibody formation by lymphocytes has been shown to significantly increase fibroblast proliferation and decrease apoptosis in the wound bed and edges [32]. Recently, eosinophils have been described as important regulators of local immunity and remodeling/repair in both health and disease, showing that eosinophils can downregulate inflammation and repair damaged tissue as well [33]. The study of the responses of this type of cells is therefore relevant in order to understand the effects of modulating agents on basic cellular biology in the wound healing process.

In the histologic inspection presented, eyes treated with the collagen matrix implant loaded with bevacizumab or the combination bevacizumab/sodium hyaluronate showed a significant reduction in inflammation and tissue cellularity, compared with eyes that were only treated with the collagen matrix implant. However, although the proposed system was able to exert an effect on cells involved in inflammation and tissue repair, this effect did not show a correlation in the clinical follow-up. This situation may be a result of the compounds being washed out of the filtering bleb before the effect was intense enough to achieve a clinical response. For the design of future studies, the improvement of the systems used in this work with biomaterials and/or compounds that are better able to control the release and effect of the drugs in the filtering bleb will be taken into consideration. We may then be able to achieve totally safe and effective control of the first stages of the scarring process.

## 5. Conclusions

In summary, this study provides a proof of concept that the localized delivery of bevacizumab in the filtering bleb decreases inflammation and lymphocyte infiltration in an animal model of glaucoma filtering surgery. Although the delivery platform of this drug still needs improvement, the results presented here show that the combination of the anti-VEGF agent with sodium hyaluronate and a collagen matrix implant could deliver bevacizumab locally to target tissues to decrease scarring and fibrosis. These data suggest that this triple combination offers new possibilities for safer adjunctive therapy, to improve the efficacy of filtration surgery and thereby the prognosis of glaucoma patients.

## Figures and Tables

**Table 1:** Mean IOP ± standard deviation values before surgery for all treatment groups (*n* = 12 eyes/group): sham-operated eyes (control), biodegradable collagen matrix implant (Olo), biodegradable collagen matrix implant loaded with bevacizumab (Olo-BVZ) and biodegradable collagen matrix implant loaded with bevacizumab, combined with sodium hyaluronate (Olo-BVZ-H5).

Treatment Group	Preoperative IOP (mmHg)
Sham	11.1 ± 0.9
Olo	11.4 ± 0.7
Olo-BVZ	11.3 ± 1.6
Olo-BVZ-H5	10.7 ± 1.1

**Table 2:** Postoperative measurements of bleb vascularity for each group shown as numbers of cases and percentages (at 24 h, 14 days and 30 days after surgery). Groups: A—control, B—Olo, C—Olo-BVZ, D—Olo-BVZ-H5. There were no significant differences between groups (*p* > 0.05).

	24 h					14 Days					30 Days				
	A	B	C	D		A	B	C	D		A	B	C	D	
Central vascularity	n (%)	n (%)	n (%)	n (%)	*p*-value	n (%)	n (%)	n (%)	n (%)	*p*-Value	n (%)	n (%)	n (%)	n (%)	*p*-Value
Avascular	0 (0)	0 (0)	0 (0)	0 (0)	0.064	0 (0)	0 (0)	0 (0)	0 (0)	0.066	1 (8)	0 (0)	0 (0)	0 (0)	0.604
Normal	0 (0)	0 (0)	0 (0)	0 (0)		6 (50)	1 (8)	1 (8)	0 (0)		5 (42)	4 (33)	4 (33)	6 (50)	
Mild	8 (67)	8 (67)	8 (67)	6 (50)		5 (42)	10 (83)	10 (83)	12 (100)		6 (50)	8 (67)	8 (67)	6 (50)	
Moderate	4 (33)	4 (33)	4 (33)	6 (50)		1 (8)	1 (8)	1 (8)	0 (0)		0 (0)	0 (0)	0 (0)	0 (0)	
Severe	0 (0)	0 (0)	0 (0)	0 (0)		0 (0)	0 (0)	0 (0)	0 (0)		0 (0)	0 (0)	0 (0)	0 (0)	
Peripheral vascularity															
Avascular	0 (0)	0 (0)	0 (0)	0 (0)	0.983	0 (0)	0 (0)	0 (0)	0 (0)	0.383	0 (0)	0 (0)	0 (0)	0 (0)	0.926
Normal	3 (25)	2 (17)	2 (17)	1 (8)		10 (83)	10 (83)	10 (83)	7 (58)		11 (92)	10 (83)	10 (83)	10 (83)	
Mild	6 (50)	8 (67)	8 (67)	9 (75)		2 (17)	2 (17)	2 (17)	5 (42)		1 (8)	2 (17)	2 (17)	2 (17)	
Moderate	3 (25)	2 (17)	2 (17)	2 (17)		0 (0)	0 (0)	0 (0)	0 (0)		0 (0)	0 (0)	0 (0)	0 (0)	
Severe	0 (0)	0 (0)	0 (0)	0 (0)		0 (0)	0 (0)	0 (0)	0 (0)		0 (0)	0 (0)	0 (0)	0 (0)	

## Data Availability

Not applicable.
